# A Multicenter, Prospective, Randomized, Double-blind Study to Evaluate the Safety and Efficacy of Saroglitazar 2 and 4 mg Compared to Pioglitazone 45 mg in Diabetic Dyslipidemia (PRESS V)

**DOI:** 10.1177/1932296813518680

**Published:** 2014-01

**Authors:** Vikas Pai, A Paneerselvam, Satinath Mukhopadhyay, Anil Bhansali, Dinesh Kamath, V Shankar, Dhiraj Gambhire, Rajendrakumar H. Jani, Shashank Joshi, Pankaj Patel

**Affiliations:** 1 Pai Clinic & Diagnostic Centre, Pune, India; 2 Aruna Diabetes Centre, Chennai, India; 3 Institute of Post Graduate Medical Education and Research (IPGME), Kolkata, India; 4 Post-Graduate Institute of Medical Education & Research (PGIMER), Chandigarh, India; 5 Sudeep Diabetes Care Centre, Bangalore, India; 6 Standard Laboratory and Polyclinic, Bangalore, India; 7 Cadila Healthcare Limited, Ahmedabad, India; 8 Joshi Clinic, Mumbai, India

**Keywords:** saroglitazar, type 2 diabetes mellitus, hypertriglyceridemia, pioglitazone

## Abstract

Dual PPARα/γ can improve both metabolic effects and minimized the side effects caused by either PPARα or PPARγ agonist. The PRESS V study was aimed to evaluate the safety, tolerability, and efficacy of saroglitazar 2 mg and 4 mg capsules (Lipaglyn™; Zydus Code: ZYH1) as compared to high dose pioglitazone in patients with diabetic dyslipidemia. In this 26-week double-blind, parallel arm, phase 3 study patients with hypertriglyceridemia with type 2 diabetes mellitus (BMI > 23 kg/m^2^; hypertriglyceridemia: TG > 200 to 400 mg/dL; glycosylated hemoglobin [HbA_1c_] >7 to 9%) were enrolled from 14 sites in India. After 2 weeks of lifestyle modification, 122 patients were randomized double-blind to 24-week treatment with the study drugs (saroglitazar 2 mg or 4 mg or pioglitazone 45 mg once daily) in a 1:1:1 ratio. The primary end point was change in plasma triglyceride level at week 24. The secondary end points were change in lipid profile and fasting plasma glucose at week 24. Patients who received study medication and had undergone at least 1 postbaseline efficacy evaluation were included in the efficacy analysis. All randomized patients who received at least a single dose were included for safety evaluation. The efficacy analysis included 109 patients (n = 37 in saroglitazar 2 mg; n = 39 in saroglitazar 4 mg; n = 33 in pioglitazone). Saroglitazar 2 mg and 4 mg significantly reduced (*P* < .001) plasma triglyceride from baseline by 26.4% (absolute change ± SD: −78.2 ± 81.98 mg/dL) and 45% (absolute change ± SD −115.4 ± 68.11 mg/dL), respectively, as compared to pioglitazone -15.5% (absolute change ± SD: −33.3 ± 162.41 mg/dL) at week 24. Saroglitazar 4 mg treatment also demonstrated marked decrease in low-density lipoprotein (5%), very-low-density lipoprotein (45.5%), total cholesterol (7.7%), and apolipoprotein-B (10.9%). Saroglitazar treatment was generally safe and well tolerated. No serious adverse events were reported in saroglitazar treatment arm and no persistent change in laboratory parameters. Saroglitazar appeared to be an effective and safe therapeutic option for improving hypertriglyceridemia in patients with type 2 diabetes mellitus.

Cardiovascular disease (CVD) is the major cause of morbidity and mortality in individuals with type 2 diabetes mellitus and responsible for 75% of deaths among type 2 diabetes patients.^1,2^ There is also 2- to 4-fold increase in cardiovascular events (coronary heart disease, stroke and peripheral vascular disease) when compared with nondiabetic patients.^3-6^ This risk is attributed to many cardiovascular risk factors including dyslipidemia and hyperglycemia.^7^ Analysis of 2 intensive glycemic control clinical studies in type 2 diabetes mellitus (Action to Control Cardiovascular Risk in Diabetes [ACCORD]^8^ and Action in Diabetes and Vascular Disease: Preterax and Doamicron Modified Release Controlled Evaluation [ADVANCE]^9^) has demonstrated an improvement in microvascular events, but not in macrovascular events including cardiovascular risk. Numerous primary and secondary prevention studies with statins have demonstrated significance lowering low-density lipoprotein cholesterol (LDL-C) in reductions in morbidity and mortality.^10-14^ Aggressive LDL-C lowering treatment has become the mainstay of lipid-lowering strategies for the last 2 decades. However despite achieving target LDL-C, a significant number of patients continue to have cardiovascular events.^15^ Recently the American Heart Association (AHA) has also identified that elevated triglyceride level has association with CVDs; however, additional outcome studies were recommended.^16^ Yet another report has estimated 9% per annum incidence of CVD despite aggressive LDL-C lowering.^17,18^

To address the microvascular and macrovascular events associated with type 2 diabetes, Zydus Research Centre has developed a dual PPAR agonist, saroglitazar. Saroglitazar is a novel dual peroxisome proliferator-activated receptor (PPAR) agonist with predominant PPAR-α and moderate -γ agonism designed to optimize a lipid and glycemic benefits with minimum effects of weight gain and edema. Preclinical and phase I/II studies have shown a favorable effect of saroglitazar on glycemic control and dyslipidemia.^19^ Prospective Randomized Efficacy and Safety of Saroglitazar (PRESS V) was designed to establish therapeutic effect of saroglitazar on triglycerides and other lipid and glucose profile with expectation of favorable safety and tolerability in type 2 diabetes.

## Methods

### Study Design and Participants

This was a prospective, randomized, double-blind, active control, interventional, phase 3 study undertaken at 14 sites in India. The study consisted of a 2-week run-in period including lifestyle modification (exercise and diet) to wash out previous medications known to affect lipid levels, followed by a double-blind treatment period for 24 weeks with either saroglitazar (2 or 4 mg capsules, Cadila Healthcare Limited, India) or pioglitazone 45 mg capsules, as an active comparator; and a follow-up visit for safety assessment at 24 week after the last treatment.

The study was good clinical practice compliant and was initiated after obtaining the approvals of the Drug Controller General of India (DCGI; F.No.12-05/05 DC, May 4, 2009), Independent/Institutional Ethics Committee (IEC) of each site, and registration of the study with Clinical Trial Registry of India (Phase III/CTRI/2009/091/000527).

Patients were recruited from July 21, 2009, to January 27, 2011, from hospital clinics and practicing physicians specialized in the treatment of diabetes. After a lifestyle modification of 2 weeks, patient had to meet the following inclusion criteria: age 18 to 65 years, high body mass index (BMI > 23 kg/m^2^), hypertriglyceridemia (fasting TG > 200 to 400 mg/dL), history of type 2 diabetes mellitus (glycosylated hemoglobin [HbA_1c_] >7% to 9%), and receiving either a sulphonylurea, metformin, or both treatments for at least 3 months. The patients were excluded if they were on insulin, glitazone or glitazar, or medications with a lipid-lowering agent in past 2 weeks, had a history of cardiac abnormalities (myocardial infarction, coronary artery bypass graft, percutaneous transluminal coronary angioplasty, unstable angina or heart failure of New York Heart Association Class III-IV), hypertension (>150/100 mmHg), thyroid dysfunction (abnormal thyroid stimulating hormone [TSH] values), hepatic dysfunction (aspartate aminotransferase/alanine aminotransferase ≥ 2.5 times of upper normal limit [UNL] or bilirubin > 2 times of UNL), gall stones, renal dysfunction (serum creatinine >1.2 mg/dL), myopathies, active muscle diseases, ketonuria, concurrent serious illness such as severe infections (tuberculosis, HIV), malignancy, alcohol or drug abuse, allergy or intolerance to the study medications, or their excipients and participation in any other clinical trial in past 3 months. In addition, pregnant female patients and nursing mothers were also excluded. All patients provided written informed consent before participation in the study.

### Procedures

At the study initiation, a diet, exercise, and lifestyle modification plan to control body weight and diabetes was discussed and implemented on the basis of investigator’s recommendation and reinforced at all the subsequent visits. After the 2-week run-in period, eligible patients were randomly assigned to double-blind treatment with 1 of the 2 doses of saroglitazar (2 mg or 4 mg capsules) or matching pioglitazone (45 mg capsules) once a day before the breakfast for 24 weeks.

Randomization was achieved by a sealed opaque envelope with a block randomization procedure to avoid an imbalance across the treatment groups. The randomization was generated by the sponsors’ statistician, who was not involved in the rest of the trial program or double-blind labeling of study drugs.

Two visits were scheduled during the run-in period: 1 for identification of the eligible patients and inviting them for lifestyle modification program and a second prerandomization visit after day 14 to confirm whether a patient met the final inclusion and exclusion criteria. Following the run-in period (2 weeks), patients visited the clinic at baseline where randomization was performed (week 0), at weeks 2, 6, 12, 18, and 24 for assessment of the safety and efficacy parameters (Table 1). Study drugs were dispensed at the baseline and weeks 2, 6, 12, and 18, and compliance was verified by pill counting.

**Table 1.** Study Schedule.

		Week						
Objective	Plan	−2	0	2	6	12	18	24
Screening	Inclusion and exclusion assessment	X						
Randomization	After 14-day lifestyle modification subjects who meet inclusion criteria		X					
Drug dispensing schedule	Drug dispensing and verification for treatment compliance		X	X	X	X	X	—
Primary efficacy	Lipid parameter: TG	X	X	—	X	X	X	X
Secondary (exploratory) efficacy	Lipid parameters: Apo A1, Apo B, HDL, LDL, TC, VLDL	X	X	—	X	X	X	X
	Glycemic indices: FPG, HbA1c	X	X	—	X	X	X	X
Safety: Clinical	Medical history, vital signs, physical examination	X	X	X	X	X	X	X
Safety: Hematology	Hemoglobin, total RBC, total WBC, differential WBC, platelet count, blood indices (PCV, MCV, MCH, MCHC)	X	X	X	X	X	X	X
Safety: LFT	AST, ALT, ALP, total bilirubin, serum proteins, total albumin and globulin, GGT	X	X	X	X	X	X	X
Safety: RFT	BUN, serum creatinine	X	X	X	X	X	X	X
Safety: Others	Uric acid, TSH, urine routine and microscopy.	X	X	—	—	—	—	X
Safety	CPK, hs-CRP	X	X	X	X	X	X	X
Cardiovascular safety	2D ECHO, ECG, USG	X	X	—	—	—	—	X

Abbreviations: ALP, alkaline phosphatase; ALT, alanine transaminase; apo A1, apo lipoprotein A1; apo B, apo lipoprotein B; AST, aspartate aminotransferase; BUN, blood urea nitrogen; CPK, creatinine phosphokinase; ECG, electrocardiogram; ECHO, echocardiogram; eGFR, estimated glomerular filtration rate; FPG, fasting plasma glucose; GGT, gamma-glutamyl transferase; HDL, high density lipoprotein; hs-CRP, high-sensitivity C-reactive protein; LDL, low density lipoprotein; LFT, liver function test; MCH, mean corpuscular hemoglobin; MCHC, mean corpuscular hemoglobin concentration; MCV, mean corpuscular volume; PCV, packed cell volume; RBC, red blood count; RFT, renal function test; TC, total cholesterol; TG, triglyceride; TSH, thyroid stimulating hormone; USG, ultrasonography; VLDL, very low density lipoprotein; WBC, white blood count.

The primary objective of this study was to confirm safety, tolerability, and efficacy of saroglitazar in the treatment of diabetic dyslipidemia. The primary end point for efficacy was absolute change in the serum triglyceride concentration from baseline to end of the treatment period. The secondary end points were changes from baseline in lipid-binding proteins (apolipoprotein A [Apo A1], apolipoprotein B [Apo B]), lipids (high-density lipoprotein cholesterol [HDL-C], LDL-C, total cholesterol [TC], very-low-density lipoprotein cholesterol [VLDL-C]), and glycemic (fasting plasma glucose [FPG], HbA_1c_) profile. Safety analysis end points were peripheral edema, cardiovascular events, body weight, and other laboratory parameters. Vital signs, physical examination, and safety laboratory assessments were measured periodically. In addition to the electrocardiographs (ECG) at baseline and weeks 2, 6, 12, 24, and 52, 2D echocardiogram (ECHO) and abdominal ultrasonography were performed at baseline, week 24, and week 52. Suspected adverse events were reviewed by the Ethics Committee, and furthermore the adverse events were adjudicated by the Data Safety Monitoring Board. All laboratory analysis was done using standard methods at Ashish Pathology Laboratory, Ahmedabad, India, which is accredited by the College of American Pathologists and the National Accreditation Board for Testing and Calibration Laboratory.

### Statistical Analysis

A minimum of 32 patients per treatment group were needed to ensure 80% power at 5% level of significance, assuming a difference of 30% between 2 groups and the common standard deviation (SD) of 42% for serum triglyceride. This estimate was derived from the previous phase II study. Assuming a 20% drop-out rate, up to 40 patients were required to be enrolled.

The primary and secondary end points were assessed by analysis of covariance (ANCOVA) to determine the least square means (LSMs) of change from baseline and 95% confidence intervals (CI). The ANCOVA model included the treatment fixed effect and baseline as covariates. Missing data were analyzed by LOCF analysis. Comparisons of each saroglitazar dose versus pioglitazone were done at a 2-sided significance level of .05. All patients who received at least 1 dose of randomized study drug and who had an evaluable baseline and at least 1 evaluable postbaseline value were included in the intention-to-treat (ITT) analysis. All patients who received at least 1 dose of a randomized study drug were included into the safety analysis.

## Results

**Figure 1.**
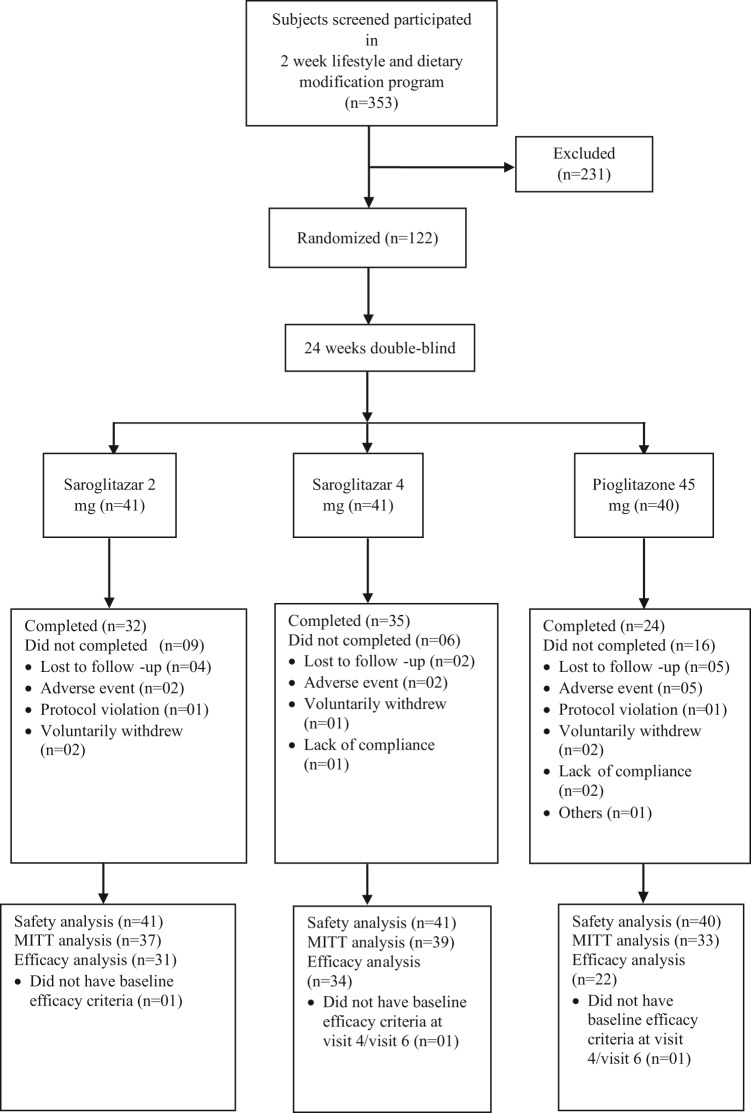
Subject disposition during the trial.

Of the 353 patients screened and participated in the 2 week lifestyle and dietary modification program, 122 patients were enrolled and randomized to 1 of the treatment groups. Disposition of subjects in this study is presented in Figure 1.

Table 2 shows that demographic characteristics and other baseline characteristics of the participants were well balanced.

**Table 2.** Baseline Demographic Characteristics and Laboratory Parameter of Participants.

	Saroglitazar 2 mg (n = 41)	Saroglitazar 4 mg (n = 41)	Pioglitazone 45 mg (n = 40)
Patients characteristics			
Female (%)	15 (36.6)	16 (39.0)	16 (40.0)
Male (%)	26 (63.4)	24 (58.5)	24 (60.0)
Age (yr)	48.9 ± 8.98	47.3 ± 9.10	49.9 ± 10.98
Weight (kg)	69.8 ± 12.72	73.0 ± 11.49	71.0 ± 12.94
Height (cm)	161.9 ± 9.44	163.1 ± 10.17	162.0 ± 10.74
BMI (kg/m^2^)	26.5 ± 3.63	27.5 ± 3.90	27.0 ± 3.72
Oral temperature (°F)	98.1 ± 0.85	98.1 ± 1.28	98.0 ± 0.90
Pulse rate (bpm)	77 ± 12.6	75 ± 11.9	77 ± 6.4
Systolic blood pressure (mm/Hg)	129 ± 8.7	129 ± 8.2	126 ± 8.1
Diastolic blood pressure (mm/Hg)	82 ± 6.2	81 ± 5.5	81 ± 4.9
Respiratory rate (/min)	17 ± 2.9	16 ± 3.0	16 ± 2.7
Laboratory data			
Hemoglobin (gm/dL)	13.6 ± 1.95	13.7 ± 1.71	13.5 ± 1.52
Triglyceride (mg/dL)	253.9 ± 68.44	257.0 ± 52.39	265.0 ± 61.66
Total cholesterol (mg/dL)	202.4 ± 47.60	197.3 ± 40.98	185.8 ± 29.91
LDL cholesterol direct (mg/dL)	134.8 ± 42.56	130.8 ± 38.83	116.6 ± 29.25
HDL cholesterol (mg/dL)	36.8 ± 12.09	35.3 ± 9.64	38.3 ± 10.85
Fasting plasma glucose (mg/dL)	143.9 ± 42.35	152.7 ± 65.99	138.2 ± 31.94
HbA1C% (mmol/mol)	8.1 ± 0.86 (65 ± 9.4 mmol/mol)	7.9 ± 0.58 (63 ± 6.3 mmol/mol)	8.2 ± 0.75 (66 ± 8.2 mmol/mol)

Data are mean ± SD, number (%) as appropriate. Abbreviations: bpm, bites per minute; BMI, body mass index; cm, centimeters; °F, degrees Fahrenheit; dL, decilitre; gm, gram; kg, kilograms; m^2^, square meters; mg, milligram; min, minute; mm/Hg, millimeter of mercury; n, number of subjects in the treatment group; yr, years.

**Figure 2.**
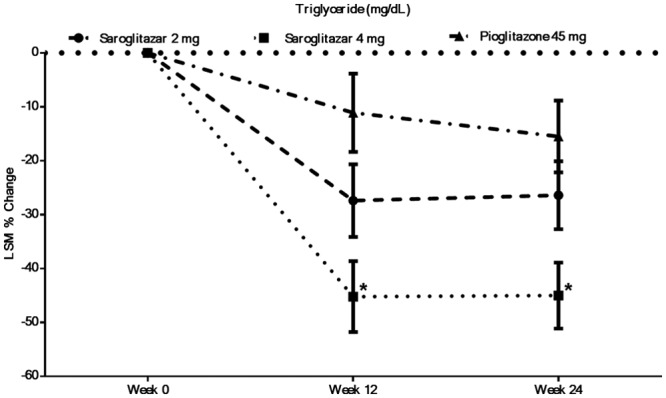
Percentage change in triglyceride level following saroglitazar and pioglitazone treatment (modified intention-to-treat population, last observation carried forward method). *Significant with respect to pioglitazone.

The effects of saroglitazar on primary and secondary efficacy end points are presented in Table 3. The primary end point of the study was the percentage change in triglyceride levels from baseline to end of the study at week 24. Saroglitazar 2 mg and 4 mg significantly decreased triglyceride levels by −27.4 ± 6.75% and −45.2 ± 6.56%, respectively, at week 12 and by −26.4 ± 31.57% and −45.0 ± 24.78%, respectively, at week 24. Triglyceride reduction was significantly better with saroglitazar 4 mg than pioglitazone 45 mg at week 24 (−45.0 ± 24.78% vs −15.5 ± 54.40%). The trend of changes over time reveals that the maximum effect of saroglitazar on triglyceride was achieved by week 12 and was sustained up to week 24 (Figure 2).

**Table 3.** Change From Baseline in Efficacy Variable at Week 24 (Modified Intention-to-Treat Population, Last Observation Carried Forward Method).

		Saroglitazar		
Efficacy parameter	Analysis	2 mg (n = 37)	4 mg (n = 39)	Pioglitazone 45 mg (n = 33)
Triglyceride (mg/dL)	Baseline mean ± SD	253.9 ± 68.44	257.0 ± 52.39	265.0 ± 61.66
	Absolute change LSM ± SD	−78.2 ± 81.98^#^	−115.4 ± 68.11* ^#^	−33.3 ± 162.41
	Percentage change LSM ± SD	−26.4 ± 31.57^#^	−45.0 ± 24.78* ^#^	−15.5 ± 54.40
LDL cholesterol direct (mg/dL)	Baseline mean ± SD	134.8 ± 42.56	130.8 ± 38.83	116.6 ± 29.25
	Absolute change LSM ± SD	3.6 ± 40.07	−12.0 ± 39.38* ^#^	3.5 ± 23.17^#^
	Percentage change LSM ± SD	12.2 ± 52.64	−5.0 ± 30.36	4.8 ± 22.58
VLDL cholesterol (mg/dL)	Baseline mean ± SD	50.3 ± 14.17	52.4 ± 12.35	55.1 ± 18.78
	Absolute change LSM ± SD	−15.2 ± 16.86^#^	−23.9 ± 15.26* ^#^	−8.8 ± 24.81^#^
	Percentage change LSM ± SD	−25.1 ± 32.93	−45.5 ± 25.12*	−20.0 ± 41.02
Total cholesterol (mg/dL)	Baseline mean ± SD	202.4 ± 47.60	197.3 ± 40.98	185.8 ± 29.91
	Absolute change LSM ± SD	2.5 ± 43.49	−18.5 ± 40.62* ^#^	9.1 ± 28.77^#^
	Percentage change LSM ± SD	5.0 ± 29.87	−7.7 ± 20.00*	5.5 ± 16.52
HDL cholesterol (mg/dL)	Baseline mean ± SD	36.8 ± 12.09	35.3 ± 9.64	38.3 ± 10.85
	Absolute change LSM ± SD	2.8 ± 11.27	0.2 ± 7.78	2.0 ± 6.86
	Percentage change LSM ± SD	12.7 ± 32.30	3.8 ± 22.11	7.1 ± 15.91
APo-A1 (mg/dL)	Baseline mean ± SD	129.4 ± 36.64	138.0 ± 30.07	137.2 ± 23.69
	Absolute change LSM ± SD	20.3 ± 58.79^#^	−2.3 ± 49.55	7.2 ± 54.86
	Percentage change LSM ± SD	27.6 ± 69.18	2.7 ± 38.86	10.0 ± 50.68
Apo-lipoproteins B (mg/dL)	Baseline mean ± SD	101.3 ± 26.77	98.3 ± 24.96	89.3 ± 18.02
	Absolute change LSM ± SD	−5.4 ± 29.96	−13.4 ± 23.41^#^	−6.4 ± 22.40
	Percentage change LSM ± SD	2.9 ± 46.79	−10.9 ± 22.32	−4.8 ± 28.90
Fasting plasma glucose (mg/dL)	Baseline mean ± SD	143.9 ± 42.35	152.7 ± 65.99	138.2 ± 31.94
	Absolute change LSM ± SD	−11.3 ± 50.11	−22.6 ± 66.30^#^	−21.8 ± 46.24
	Percentage change LSM ± SD	−1.5 ± 39.42	−8.3 ± 31.91	−12.8 ± 30.06
HbA1c (%)	Baseline mean ± SD	8.1 ± 0.86	7.9 ± 0.58	8.2 ± 0.75
	Absolute change LSM ± SD	−0.3 ± 0.83^#^	−0.3 ± 0.60^#^	−0.4 ± 0.72^#^

Abbreviations: dL, deciliter; LOCF, last observation carried forward; LSM, least square mean; mg, milligram; SD, standard deviation; SE, standard error.

* Significant compared to pioglitazone. ^#^Significant compared to baseline.

After 24 weeks treatment with saroglitazar 4 mg, there was significant absolute mean decrease in LDL-C (−12.0 ± 39.38 mg/dL), VLDL-C (−23.9 ± 15.26 mg/dL), TC (−18.5 ± 40.62 mg/dL), Apo B (−13.4 ± 23.41 mg/dL), FPG (−22.6 ± 66.30 mg/dL), and HbA_1c_ (−03 ± 0.60%) compared to baseline. Saroglitazar 4 mg was statistically superior to pioglitazone 45 mg in reducing LDL-C (saroglitazar −12.0 ± 39.38 mg/dL vs pioglitazone 3.5 ± 23.17 mg/dL), VLDL-C (saroglitazar −23.9 ± 15.26 mg/dL vs pioglitazone −8.8 ± 24.81 mg/dL), TC (saroglitazar −18.5 ± 40.60 mg/dL vs pioglitazone 9.1 ± 28.77 mg/dL). Saroglitazar and pioglitazone had similar effects on HDL-C, apolipoprotein A1, and apolipoprotein B. As far as lipid profile of diabetic dyslipidemia is concerned, numerically more number of patients has achieved ATP III goals with saroglitazar 4 mg than in pioglitazone arm (Table 4).

**Table 4.** Assessment of ATP Goal Following Saroglitazar.

ATP goal (week 24 per protocol)^a^	Saroglitazar 4 mg (%) (n = 34)	Pioglitazone 45 mg (%) (n = 22)
Not achieved even 1 criterion	29.4	50.0
Achieved 1 criterion	26.5	22.7
Achieved 2 criteria	35.3	27.3
Achieved all 3 criteria	8.8	0.0

a Male: triglyceride < 150 mg/dL, LDL < 100 mg/dL, HDL > 40 mg/dL.

Female: triglyceride < 150 mg/dL, LDL < 100 mg/dL, HDL > 50 mg/dL.

Saroglitazar 2 mg and 4 mg and pioglitazone 45 mg showed comparable effects in reducing FPG and HbA_1c_ at week 24.

There were no significant changes from baseline and between the treatment arms in any of the safety laboratory findings. There was mean increase of 1.6 kg in pioglitazone arm and no significant change in bodyweights in saroglitazar arms (Table 5). In all, 7 of 41 patients reported adverse events in the saroglitazar 2 mg and 4 mg arms, while 11 of 40 patients reported adverse events in the pioglitazone group. The most frequently reported adverse events were asthenia, gastritis, chest discomfort, peripheral edema, dizziness, and tremors (Table 6). Most of adverse events were considered unrelated to treatment and were of mild intensity. There was no serious adverse event in the saroglitazar arms. There was also no clinically significant change in ECG, 2D ECHO, or USG findings in any of the treatment groups. Two patients had SAEs in the pioglitazone treatment arm; 1 had suspected acute myocardial infarction and another had hematemesis. The suspected case of myocardial infarction was declared dead on arrival at the hospital, and no investigation could be performed. Another patient was hospitalized, treated, and discharged without any other sequelae. The investigators and DSMB have adjudicated these SAEs as nontreatment emergent.

**Table 5.** Assessment of Safety Laboratory Parameters at Week 24 (Modified Intention-to-Treat Population).

		Saroglitazar	Saroglitazar	Pioglitazone
Analysis	Safety parameter	2 mg (n = 37)	4 mg (n = 39)	45 mg (n = 33)
Hemoglobin (gm/dL)	Baseline mean ± SD	13.6 ± 1.95	13.7 ± 1.71	13.5 ± 1.52
	Absolute change mean ± SD	−0.0 ± 0.06	−0.0 ± 0.08	−0.0 ± 0.11
MCH (pg)	Baseline mean ± SD	27.1 ± 2.99	27.8 ± 2.15	27.3 ± 3.70
	Absolute change mean ± SD	0.0 ± 0.05	0.0 ± 0.06	0.1 ± 0.42
MCHC (g/dL)	Baseline mean ± SD	29.5 ± 2.43	29.8 ± 2.39	29.6 ± 2.21
	Absolute change mean ± SD	0.0 ± 0.09	−0.0 ± 0.08	0.0 ± 0.17
MCV (fL)	Baseline mean ± SD	91.8 ± 9.21	93.8 ± 8.54	92.2 ± 11.24
	Absolute change mean ± SD	0.0 ± 0.08	0.0 ± 0.08	0.1 ± 0.17
PCV (%)	Baseline mean ± SD	46.1 ± 6.09	45.9 ± 5.69	45.8 ± 5.84
	Absolute change mean ± SD	−0.0 ± 0.1	−0.0 ± 0.12	−0.0 ± 0.13
Total leucocyte count (10^3/uL)	Baseline mean ± SD	8.5 ± 2.48	7.8 ± 1.73	8.2 ± 2.33
	Absolute change mean ± SD	−0.1 ± 0.16	−0.0 ± 0.31	−0.1 ± 0.16
Total platelet count (10^3/uL)	Baseline mean ± SD	248.6 ± 74.76	255.9 ± 73.99	281.3 ± 99.73
	Absolute change mean ± SD	0.1 ± 0.21	0.0 ± 0.24	0.0 ± 0.25
Total RBC (10^6/uL)	Baseline mean ± SD	5.0 ± 0.52	4.9 ± 0.53	5.0 ± 0.71
	Absolute change mean ± SD	−0.0 ± 0.08	−0.0 ± 0.12	−0.1 ± 0.19
Thyroid stimulating hormone (mIU/L)	Baseline mean ± SD	2.4 ± 1.35	2.5 ± 1.55	2.8 ± 2.35
	Absolute change mean ± SD	−0.4 ± 0.19	−0.2 ± 0.26	0.7 ± 1.25
Albumin (g/dL)	Baseline mean ± SD	4.5 ± 0.30	4.5 ± 0.28	4.5 ± 0.30
	Absolute change mean ± SD	0.0 ± 0.08	0.0 ± 0.06	−0.0 ± 0.05
Globulin (g/dL)	Baseline mean ± SD	2.8 ± 0.44	2.9 ± 0.56	2.9 ± 0.50
	Absolute change mean ± SD	0.0 ± 0.15	0.0 ± 0.20	−0.0 ± 0.13
Protein total (g/dL)	Baseline mean ± SD	7.3 ± 0.49	7.4 ± 0.54	7.5 ± 0.53
	Absolute change mean ± SD	0.0 ± 0.09	0.0 ± 0.08	−0.0 ± 0.07
Alkaline phosphates (U/L)	Baseline mean ± SD	81.9 ± 24.93	85.0 ± 31.78	84.1 ± 26.57
	Absolute change mean ± SD	−0.2 ± 0.28	−0.2 ± 0.56	−0.1 ± 0.24
ALT (U/L)	Baseline mean ± SD	31.5 ± 16.48	29.7 ± 15.91	26.3 ± 9.13
	Absolute change mean ± SD	−0.1 ± 0.36	−0.2 ± 0.30	−0.2 ± 0.25
AST (U/L)	Baseline mean ± SD	25.9 ± 15.75	23.6 ± 9.69	22.1 ± 5.81
	Absolute change mean ± SD	0.2 ± 0.63	0.1 ± 0.43	0.0 ± 0.42
GGTP (U/L)	Baseline mean ± SD	37.6 ± 22.85	35.3 ± 18.75	36.4 ± 22.86
	Absolute change mean ± SD	−0.2 ± 0.40	−0.3 ± 0.43	−0.3 ± 0.25
Bilirubin (mg/dL)	Baseline mean ± SD	0.5 ± 0.20	0.5 ± 0.34	0.5 ± 0.2
	Absolute change mean ± SD	−0.2 ± 0.32	−0.0 ± 0.54	0.1 ± 0.85
Creatinine (mg/dL)	Baseline mean ± SD	0.7 ± 0.21	0.7 ± 0.19	0.7 ± 0.2
	Absolute change mean ± SD	0.1 ± 0.26	0.2 ± 0.44	0.0 ± 0.2
BUN (mg/dL)	Baseline mean ± SD	10.8 ± 3.66	9.5 ± 2.75	11.1 ± 2.74
	Absolute change mean ± SD	0.1 ± 0.28	0.2 ± 0.47	0.2 ± 0.37
Uric acid (mg/dL)	Baseline mean ± SD	5.0 ± 1.32	5.0 ± 1.76	4.6 ± 1.22
	Absolute change mean ± SD	−0.1 ± 0.17	0.0 ± 0.11	−0.3 ± 0.56
CPK (U/L)	Baseline mean ± SD	91.3 ± 62.48	96.3 ± 49.4	97.2 ± 47.82
	Absolute change mean ± SD	0.3 ± 0.94	0.3 ± 0.49	0.3 ± 0.46
hs-CRP (mg/L)	Baseline mean ± SD	3.1 ± 3.23	4.5 ± 5.31	3.3 ± 3.37
	Absolute change mean ± SD	0.6 ± 2.11	0.2 ± 1.61	0.1 ± 1.43
Body weight (kg)	Baseline mean ± SD	69.8 ± 12.72	73.0 ± 11.49	71.0 ± 12.94
	Absolute change mean ± SD	−0.8 ± 5.35	−0.1 ± 2.70	1.6 ± 3.44

Abbreviations: ALT, alanine transaminase; AST, aspartate aminotransferase; BUN, blood urea nitrogen; CPK, creatinine phosphokinase; dL, deciliter; GGTP, gamma-glutamyltransferase; gm, gram; hs-CRP, high-sensitivity C-reactive protein; kg, kilogram; L, liter; MCH, mean corpuscular hemoglobin; MCHC, mean corpuscular hemoglobin concentration; MCV, mean corpuscular volume; mg, milligram; PCV, packed cell volume; pg, pictograms; RBC, red blood count; SD, standard deviation; U/L, unit per liter.

**Table 6.** Adverse Event Reported for 3 or >3 Patients in the Study.

	Saroglitazar 2 mg (n = 41)	Saroglitazar 4 mg (n = 41)	Pioglitazone 45 mg (n = 40)
Adverse event	Number of patients (percentage)		
Asthenia	1 (2.4)	3 (7.3)	1 (2.5)
Gastritis	0 (0.0)	2 (4.9)	2 (5.0)
Chest discomfort	1 (2.4)	1 (2.4)	1 (2.5)
Peripheral edema	1 (2.4)	0 (0.0)	2 (5.0)
Dizziness	1 (2.4)	1 (2.4)	1 (2.5)
Tremors	1 (2.4)	1 (2.4)	1 (2.5)

## Discussion

Prospective Randomized Efficacy and Safety of Saroglitazar (PRESS V) is the first prospective confirmatory clinical study of saroglitazar, a novel dual PPARα/γ agonist, in diabetic dyslipidemia. In this 24 week study, saroglitazar has produced dose-related decrease in triglyceride level at 2 mg and 4 mg. Effects of saroglitazar 2 mg and 4 mg, on triglycerides was significantly better than baseline. It has shown 45% reduction in triglycerides, which was comparable to triglyceride reduction of 43% reported with aleglitazar, another dual PPAR agonist.^20^ It has been reported that fenofibrate, a PPARα agonist, decreases triglyceride level by 28.9% and 35.9%, when baseline values were 191 mg/dL and 231.9 mg/dL, respectively.^21^ Although it is difficult to compare clinical studies conducted in different setting, it seems that saroglitazar has similar or improved effect on triglyceride compared to other antidyslipidemic agents, either in development or in market. Our study is exceptionally small in comparison to previously reported study, where pioglitazone and rosiglitazone were compared for lipid and glycemic effects in patients with diabetic dyslipidemia.^22^ The data revealed that pioglitazone was associated with significant improvement in triglyceride (percentage change: −12), HDL cholesterol (percentage change: 14.9), non-HDL cholesterol (percentage change: 3.8), and LDL particle concentrations and LDL particle size apart from glycemic control. In this study pioglitazone has shown −15.5% decreases in triglyceride level and other parameters were also in the line of previously reported data.^22^

Saroglitazar 2 mg and 4 mg have also demonstrated significant and better reduction in absolute mean values of other atherogenic lipids, that is, LDL-C, VLDL-C, TC, Apo B as compared to baseline. Its effect on atherogenic lipids contributes toward protection against cardiovascular risk.

The PPAR-γ agonist pioglitazone is used in the treatment of diabetes and by virtue of its favorable effects on lipid profile; it is expected to have positive effect on cardiovascular complications. Higher numbers of patients achieved the primary lipid goals as per ATP III criteria in saroglitazar arms as compared to pioglitazone arm.

Saroglitazar 2 and 4 mg also have shown a dose-related decrease in FPG. They also improved HbA_1c_ level. Saroglitazar 4 mg was comparable to pioglitazone 45 mg as far as FPG and HbA_1c_ reductions are concerned. The decrease in HbA_1c_ level was lesser in all treatment arms compared; however, the reduction also depends on the baseline value; the higher the value, the greater the decrease. An observational, open-label efficacy and safety study of pioglitazone in Indian type 2 diabetes mellitus patients also showed similar results after 6 months of treatment.^23^ Aleglitazar 150 µg was also found to be similar with pioglitazone 45 mg in reducing the HbA_1c_ and fasting glucose levels.^20^

Considering antidyslipidemic and antiglycemic effects, saroglitazar has the potential to address the challenges of reduction of macrovascular and microvascular events in larger outcome studies. However, further prospective research is required to establish this fact.

In addition, saroglitazar seems to be safe and well tolerated over a course of 24 weeks. Excessive cardiovascular events have also been noted for patients given muraglitazar and rosiglitazone following a short therapy.^24,25^ The sample size of this study was small to make definitive conclusions, but no cardiovascular events occurred, which is reassuring.

Monitoring of body weight and peripheral edema also suggested that these side effects were absent in saroglitazar 4 mg but reported with pioglitazone. Pioglitazone arm also has been shown to increase body weight unlike saroglitazar arms. Changes in serum creatinine values were not significant compared to baseline and also pioglitazone arm. Although long-term improvement in lipid profile with saroglitazar is yet to be studied, the difference noted between the 2 drugs is substantial and likely to provide benefits for cardiovascular outcomes in patients with type 2 diabetes. However, further research is recommended.

The broad range of lipid improvements associated with saroglitazar demonstrates the potential of saroglitazar to address the pattern of dyslipidemia very often seen in patients with type 2 diabetes, which includes high levels of triglycerides, low levels of HDL-C, and a moderate increase in LDL-C. Although the higher cholesterol is the most recognized primary target of lipid-lowering therapy in diabetes, correction of hypertriglyceridemia and low concentration of HDL-C are likely to address the issue of residual risk,^26^ and further reduction in cardiovascular event.^27^ Although the present study was designed to recruit patients who would as closely as possible represent a population with type 2 diabetes. However, there are limitations to the generalization of these data. Exclusion criteria resulted in enrollment of patients who were not at high risk of CVD. Patients generally were taking metformin or sulfonylurea or both the medications at the time of enrollment. The study did not allow changes or dose titrations of antidiabetic drugs during the study period. With this background, we consider that antidyslipidemic and antidiabetic effects seen during the study could be attributed to saroglitazar. During the study, no antidyslipidemic agents were permitted. The 24-week course of therapy in the present study was regarded as a standard duration for a phase 3 study; however, 24 weeks is short to show long-term cardiovascular safety and tolerability of saroglitazar. The absence of any safety signals during the study and also up to 24 weeks posttreatment is encouraging for the use of saroglitazar in long-term studies.

## Conclusions

The significant changes in lipids and glycemic end points with favorable safety profile represent promising data for saroglitazar to grant marketing permission for the treatment of diabetic dyslipidemia. Long-term phase 4 studies with saroglitazar have been initiated by the sponsor to further elucidate its efficacy and safety in dyslipidemic patients.
